# On How Definitions of Habits Can Complicate Habit Research

**DOI:** 10.3389/fpsyg.2019.02642

**Published:** 2019-11-29

**Authors:** Jan De Houwer

**Affiliations:** Department of Experimental Clinical and Health Psychology, Ghent University, Ghent, Belgium

**Keywords:** habits, automaticity, conceptual analysis, learning, goal-directed behavior

## Abstract

The core message of this paper is that many of the challenges of habit research can be traced back to the presence of causal elements within the definition of habits. For instance, the idea that habits are stimulus-driven implies that habitual behavior is not causally mediated by goal-representations. The presence of these causal elements in the definition of habits leads to difficulties in establishing empirically whether behavior is habitual. Some of these elements can also impoverish theoretical thinking about the mechanisms underlying habitual behavior. I argue that habit research would benefit from eliminating any reference to specific S-R association formation theories from the definition of habits. Which causal elements are retained in the definition of habits depends on the goals of researchers. However, regardless of the definition that is selected, it is good to be aware of the implications of the definition of habits for empirical and theoretical research on habits.

When asked to explain their behavior, lay people often refer to habits. Likewise, when making resolutions for the future, they often express a wish to install new habits or to change old ones. The concept “habit” is popular not only with lay people but also engages academic psychologists (see Wood and Rünger, 2016, for a review). As noted by Gardner (2015), one important difference in the way habits are conceptualized by lay people versus academics is that the former focus on observable aspects of behavior (e.g., the frequency with which a behavior is emitted) whereas the latter focus on the (mental) causes of behavior (e.g., the fact that the behavior is triggered by cues in the environment without being directed at goals; see Wood and Neal, 2007, for a discussion of the interface between habits and goals).

Although the focus on explanation is an undeniable strength of the academic approach to habits, in this paper, I draw attention the downsides of incorporating assumptions about causes into definitions of habits and habitual behavior. In the section “The Conceptual Level: Defining Habits and Habitual Behavior,” I briefly consider some of the definitions of habits that have been put forward by lay people and academics. These definitions have in common that they have implications for the criteria that are used to distinguish empirically between habitual and non-habitual behavior. In the section “The Empirical Level: Establishing the Presence of Habitual Behavior,” I discuss problems with empirically verifying the causal criteria put forward in the scientific literature on habits. The section “The Theoretical Level: Explaining Habitual Behavior” focuses on the constraints in theorizing that follow from definitions of habits that refer to S-R associations (i.e., links between stimulus and response representations *via* which activation can spread). Finally, I discuss the possible merits of removing causal assumptions from the definition of habits. Many of the challenges that are addressed in this paper have been discussed before by others (e.g., Watson and de Wit, 2018). The current paper aims to go beyond those past contributions by highlighting how these challenges relate to the causal nature of scientific definitions of habits. Based on this insight, new ways of tackling these challenges can be considered.

## The Conceptual Level: Defining Habits and Habitual Behavior

The first challenge for any area of research is to reach some level of clarity about and consensus on what is being studied (i.e., what constitutes the explanandum). To the extent that definitions of a research topic diverge, scientific progress is bound to be hampered by misunderstandings and false debates. Although the definition of a concept can change over time and general agreement about definitions is rare in psychological science, there is merit in trying to improve clarity at the conceptual level, if only by creating awareness of the various definitions that have been proposed and the way in which they are related (Machado and Silva, 2007). In this section, I will first consider the different ways in which habits have been defined. This allows me to then highlight the causal nature of those definitions and the implications this has for habit research.

The recent paper of Gardner (2015) provides an excellent starting point for considering the range of definitions of habits that have been proposed. Lay definitions are mainly descriptive, referring to habits as behaviors that are emitted frequently or in a persistent, automatic manner. Scientific definitions of habits, on the other hand, contain explanatory elements. Some of these scientific definitions also refer to habits as (frequent, persistent, or automatic) behaviors but in addition those behaviors are said to have particular causes. These causes can refer to past experience, such as the idea that habits are the result of the repetition of behavior, and/or to underlying mental processes, such as the idea that habits result from the activation of S-R associations without the involvement of goals (i.e., representations of desired end states; see Gardner, 2015, for an overview). Many of these definitions imply that habitual behavior is stimulus-driven, that is, dependent on cues in the current context that trigger the behavior without considerations of the current outcomes of the behavior^1^. Other scientific definitions do not refer to habits as a behavior but as a mental cause underlying behavior. For instance, habits have been defined as behavioral impulses that are instigated by S-R associations or as the S-R associations themselves. Many definitions, however, refer to several of these components. To illustrate, Gardner et al. (2011, p. 175) define habits as “behavioural patterns learned through context dependent repetition: repeated performance in unvarying settings reinforces context-behaviour associations such that, subsequently, encountering the context is sufficient to automatically cue the habitual response.” Wood and Neal (2009, p. 580) define habits as “A type of automaticity characterized by a rigid contextual cuing of behavior that does not depend on people’s goals and intentions. Habits develop as people respond repeatedly in a stable context and thereby form direct associations in memory between that response and cues in the performance context.”

Gardner (2015) already highlighted the fundamental difference between habits as a type of behavior and habits as a underlying determinant of behavior (e.g., an impulse or S-R association). To reduce confusion, in this paper, I will use the term “habitual behavior” to refer to habits as a type of behavior. Importantly, all definitions of habits put forward criteria for distinguishing between habitual and non-habitual behavior. These criteria can refer to more or less observable characteristics of behavior (e.g., frequency, persistence, automaticity); to assumptions about the experiences that cause this behavior (e.g., repetition of a behavior in a context); and/or to assumptions about the mental processes and representations that cause the behavior (e.g., the activation of an impulse *via* the operation of an S-R association).

In this paper, I focus on the implications of the criteria that habit researchers use to distinguish habitual from non-habitual behavior. Although Gardner (2015) correctly points out that scientists should move beyond mere description of behavior and consider the causes of behavior, there are downsides to incorporating causal elements within scientific definitions of to-be-explained phenomena. First, it can hamper attempts to verify empirically whether the phenomenon is present (i.e., to determine whether a behavior qualifies as habitual), which leads to difficulties in studying the phenomenon. Causality can never be observed directly but must always be inferred from observable events. This problem is exacerbated when the causes themselves are unobservable, as is the case with many mental processes and representations (e.g., S-R associations in memory). Second, defining phenomena in terms of their causes confounds the explanandum (that which needs to be explained) with the explanans (that by which the explanandum is explained; Hempel, 1970). In other words, it implies *a priori* assumptions about the causes of the phenomenon. This is less problematic when those *a priori* assumptions turn out to be justified. However, if those assumptions are incorrect, then research based on this definition does not necessarily inform us about the phenomenon, thus hampering the cumulative nature of research. Moreover, an *a priori* commitment to certain causes of a phenomenon may prevent researchers from considering the role of other potential causes of the phenomenon, thereby reducing theoretical diversity and ultimately hampering theoretical progress.

In the remainder of this paper, I discuss these challenges at the empirical and theoretical level, as well as possible ways to deal with those challenges. Rather than providing a systematic review of the literature in order to assess the exact extent to which problems at the empirical and theoretical level arise in habit research, I will focus on developing the conceptual argument and will merely provide examples of the problems that can arise. The examples that I provide come from behavioral research on habits in humans. The conceptual issues that I address also apply to neuroscientific research on habits in humans but this research will not be covered in this paper.

## The Empirical Level: Establishing the Presence of Habitual Behavior

For many psychologists, the defining characteristic of habits is that they are stimulus-driven (Gardner, 2015; Wood and Rünger, 2016). This idea introduces several causal assumptions within the definition of habitual behavior. In the following paragraphs, I will highlight these causal assumptions, as well as the challenges they create for establishing that behavior is habitual in the sense of stimulus-driven.

On the one hand, the concept of stimulus-driven behavior implies that habitual behavior is caused directly by stimuli in the environment. Although the causal impact of stimuli on behavior cannot be observed directly, it is relatively easy to infer the environmental causes of behavior by manipulating the presence of stimuli and examining how this influences the presence of the behavior. If the behavior is present when a certain stimulus is present in the environment but absent when that stimulus is absent, this provides strong grounds for arguing that the stimulus is causally related to the occurrence of the behavior.

On the other hand, in the context of habit research, “stimulus-driven” not only implies that a stimulus is causally related to the behavior but also that the behavior is not a function of its anticipated consequences. Put differently, stimulus-driven behavior is not directed at goals (Adams, 1982; Heyes and Dickinson, 1990; see Moors et al., 2017, for a detailed analysis of what it means to say that behavior is goal-directed). Hence, establishing that a behavior is habitual requires arguments for the conclusion that the behavior is not directed at goals^2^. There are, however, several reasons why it is not easy to convincingly demonstrate that behavior is not directed at goals. First, goal representations are mental entities that cannot be observed directly by researchers (and, in the case of unconscious goals, also not by the person who possesses the goal). Second, whether these entities have a causal impact can also not be observed directly because causality always needs to be inferred from observations. Third, verifying the absence of causal impact of mental entities is even more difficult to achieve than verifying the presence of these entities and their causal impact on behavior.

Habit researchers have tried to circumvent the first two problems by using behavioral proxies of the causal impact of goal representations on behavior. They reasoned that if a goal causally mediates a behavior, then changing the goal or its relation to the behavior should also change the behavior. For instance, in order to establish that lever pressing is mediated by the goal to eat a specific food, one could reduce the goal to eat that food by making it aversive (i.e., devaluation test) or by no longer delivering the food after a lever press (i.e., contingency degradation test). From a cognitive point of view, it is indeed relatively safe to conclude that the behavior is mediated by a particular goal representation if those interventions change behavior (e.g., Adams, 1982; Heyes and Dickinson, 1990).

Whereas this strategy might circumvent the first two problems that were noted above, it does not solve the third problem. More specifically, behavior may be mediated by goals even if an effect of devaluation and contingency degradation is not found (Heyes and Dickinson, 1990; Thrailkill and Bouton, 2015; Moors et al., 2017). It is indeed possible that the intervention was not strong enough (e.g., it did not fully eliminate the palatability of the food), that statistical power was insufficient for establishing the presence of goals and their impact on behavior (see Vadillo et al., 2019), or that the intervention targeted another goal than the one that actually mediates behavior (De Houwer et al., 2018).

With regard to the latter point, De Houwer et al. (2018) examined one of the most widely used paradigms in research on habits in humans, namely the fabulous fruit game (e.g., de Wit et al., 2007). Without going into detail, in this task, participants repeatedly press keys in order to generate images of fruits, some of which are worth points. During an outcome devaluation phase, the value of some of the fruits is reduced (i.e., they are no longer worth points). Habits are typically inferred from the lack of impact of fruit-devaluation on key presses. However, the data reported by De Houwer et al. support the idea that these seemingly habitual key presses are still directed at the goal of obtaining points. For instance, changing the value of points did influence responding even when changing the value of fruits did not.

As another example, consider the well-known study of Neal et al. (2011). These authors observed that people who often eat popcorn when watching a movie in a cinema theater (“habit” group) will continue to eat popcorn even when it is stale (i.e., devalued) whereas people who do not often eat popcorn in cinemas (“nonhabit” group) stop eating stale popcorn. Although this suggests that eating popcorn in the “habit” group is not mediated by the goal to have tasty food whereas that goal does mediate popcorn eating in the “nonhabit” group, it does not necessarily imply that popcorn eating in the “habit” group was stimulus-driven. For instance, it is possible that eating popcorn in the “habit” group was mediated by the goal to have a more complete cinematic experience. Let us assume that for people who often eat popcorn in a cinema theater, the cinematic experience is not complete without eating popcorn whereas for controls, the richness of the cinematic experience does not depend on eating popcorn. If this assumption is correct, then eating stale popcorn will be goal-conductive for members of the “habit” group but not for controls. In other words, people in the “habit” group might be more willing to tolerate the bad taste of the stale popcorn because for them, eating popcorn while watching a movie has merit as such, even when it does not taste good. Of course, it remains to be seen whether these auxiliary assumptions about the differences in the goal-conduciveness of eating popcorn in the “habit” and “nonhabit” group are valid. If additional studies do not provide support for the alternative goal-directed account, one should be willing to accept the conclusion that the behavior is habitual rather than adhere to the irrefutable claim that the behavior must be mediated by some type of goal. Nevertheless, researchers should consider the possibility that devaluation and contingency degradation tests lack sensitivity or fail to target the goal that is actually driving behavior (De Houwer et al., 2018)^3^.

These problems cannot be sidestepped by inferring the lack of goal-directedness from the automatic nature of behavior. Because stimulus-driven behavior is assumed to be automatic, one might see evidence for automaticity as an indication of the fact that behavior is stimulus-driven. However, it is now generally accepted that automatic behavior is not necessarily stimulus-driven (e.g., Bargh, 1989, 1990; Aarts and Dijksterhuis, 2000; for a recent discussion, see Huang and Bargh, 2014). Even addictive behaviors, which are often seen as prototypical examples of automatic behavior because they are emitted despite their obvious negative consequences, are now considered by some to be directed at realizing goals (e.g., Baumeister, 2017; Hogarth, 2018; Kopetz et al., 2018). Moreover, if one would decide to infer the stimulus-driven nature of behavior from its automaticity, there remains the problem of establishing whether behavior qualifies as automatic. Just like there are many definitions of the concept “habit,” there are many definitions of the concept “automatic.” Most of these definitions refer to one or more automaticity feature, such as unintentional, involuntary, fast, efficient, or unconscious (Bargh, 1989, 1994; Moors and De Houwer, 2006). Because different automaticity features do not necessarily co-occur, establishing automaticity thus requires one to specify the automaticity features one has in mind and to test the presence of each feature individually. This opens up debates about which features are crucial for determining whether a behavior is automatic and whether the term “automaticity” is still useful as a unifying concept (Fiedler and Hütter, 2014). Moreover, Moors (2016) convincingly argued that the extent to which a type of behavior or process displays a certain feature of automaticity (e.g., the extent to which semantic processing depends on conscious input) can vary across contexts. Hence, there is little merit in saying that a process or behavior has a certain feature of automaticity in an absolute sense (e.g., that semantic processing *is* an unconscious process)^4^. For all these reasons, there is little merit in establishing the stimulus-driven nature of behavior on the basis of its automaticity.

Many definitions of habits do not only incorporate the assumption that habits are stimulus-driven but also assumptions about the factors that are responsible for the stimulus-driven nature of habits (see Gardner, 2015). First, it is often assumed that behavior becomes stimulus-driven if it has been frequently emitted in the context of a certain stimulus. Second, many researchers assume that stimulus-driven behavior is instigated *via* the activation of S-R associations that have been formed gradually as the result of the frequent co-occurrence of a stimulus and a behavior. Although both proposals certainly have merits, from the current perspective, they add additional causal elements to the definition of habits and habitual behavior which result in additional difficulties in establishing whether behavior qualifies as habitual.

Let us first consider frequency as a cause of stimulus-driven behavior. Many researchers assume that behavior should become more habit-like (i.e., stimulus-driven) the more frequently it is emitted in a certain context, that is, the more it is overtrained (e.g., de Wit et al., 2018). The causal role of frequency can be examined by manipulating the frequency of a behavior and observing indices of the stimulus-driven nature of the behavior. Note, however, that experimental designs allow for causal conclusions only if confounding variables are controlled for. For instance, the frequency of behavior (i.e., how often it occurs) can be confounded with the recency of behavior (i.e., the time elapsed between the test phase and the most recent occurrence of a behavior). Assuming that the impact of past events decreases with time and/or the number of intervening events (Ebbinghaus, 1913), it is possible that differences at test between frequent and infrequent behavior reflect recency rather than frequency or a combination of both. A confound between frequency and recency is typically avoided by varying frequency while keeping recency constant across conditions (e.g., de Wit et al., 2018). Another approach which has been implemented less frequently in the literature on habits is to manipulate frequency and recency orthogonally so that the relative contribution of and interaction between both factors can be examined (e.g., Schmidt et al., 2019, submitted).

Researchers often also refer to S-R associations as the mental cause of stimulus-driven behavior. More specifically, they assume that a stimulus can initiate a behavior by activating its representation in memory, activation which can then spread *via* the S-R association to the response representation and thereby bring about the response without the involvement of the representations of goals. Traditionally, S-R associations are assumed to form gradually as the result of the frequent co-occurrence of a stimulus and a behavior, as well as the presence of rewards that follow the behavior when it is emitted in the presence of the stimulus (e.g., de Wit et al., 2007; Wood and Rünger, 2016). Establishing that stimulus-driven behavior is mediated by S-R associations is faced with the same problems as establishing stimulus-driven behavior (see above) but also with the additional problem of demonstrating the mediating role of S-R associations. The fact that neither S-R associations themselves nor their causal impact can be observed directly complicates efforts to verify the involvement of S-R associations in behavior. Procedures have been developed to assess the strength of S-R associations indirectly (e.g., *via* their impact on lexical decision times; e.g., Neal et al., 2012) but the usefulness of these procedures depends on how valid they are (see De Houwer, 2011).

One could argue that stimulus-driven behavior is by definition mediated by S-R associations and that evidence for the stimulus-driven nature of behavior thus constitutes evidence for the mediating role of S-R associations. However, in that case, it is not clear what the idea of S-R associations adds to the notion of stimulus-driven behavior. Such added value can come only from specific theoretical ideas about what those S-R associations are (e.g., abstractive links between mental representations of stimuli and responses), how they are formed (e.g., gradually as the result of repetition or rewards), and how they influence behavior (e.g., *via* the automatic spreading of activation from the stimulus to the associated response representation; e.g., de Wit et al., 2007). Hence, incorporating the notion of S-R associations into the definition of habits comes with specific theoretical commitments, which requires the specification of additional criteria to distinguish “real” habitual behavior (i.e., stimulus-driven behavior that is mediated by a specific type of S-R associations that develops under specific conditions) from other stimulus-driven behavior (i.e., behavior that is stimulus-driven but mediated by another type of representation). In sum, defining habits in terms of S-R associations only aggravates the problem of empirically verifying whether behavior is “truly” habitual.

## The Theoretical Level: Explaining Habitual Behavior

Introducing causal elements within the definition of habits and habitual behavior not only results in challenges at the empirical level (i.e., the possibility of verifying that behavior is habitual) but can also limit theoretical innovation. In this section, I focus primarily on definitions of habits that refer to S-R associations. After discussing their dominance, I sketch two alternative theories of habitual (in the sense of stimulus-driven) behavior. In that way, I hope to clarify that it is not only possible to consider other models when trying to explain habitual behavior but also that it can be beneficial to do so. Considering these other models is, however, only possible if one removes the notion of S-R associations from the definition of habits.

The current theoretical literature on habits is dominated by S-R association formation models. Even researchers who do not explicitly define habits in terms S-R associations often consider only S-R associations when trying to explain habitual behavior (e.g., Wood and Rünger, 2016). This dominance of S-R association models in the literature on habits is probably based on the fact that these models are compatible with the widespread definition of habitual behavior as behavior that is automatic and stimulus-driven as the result of frequent stimulus–response co-occurrences. Behavior that is driven by S-R associations (1) must have been emitted frequently enough to allow for the gradual formation of an S-R association, (2) has features of automaticity because activation can spread across associations automatically, and (3) is stimulus-driven in that the activation of S-R associations is instigated only by a stimulus and does not involve goal representations. In fact, the match between the mechanism of activating gradually acquired S-R associations and the phenomenon of habitual (i.e., frequency-induced automatic stimulus-driven) behavior is so good that one might wonder whether any other mechanism could account for behavior that is stimulus-driven and automatic. In this section, I briefly discuss two of these alternatives just to illustrate that (1) other mechanisms are possible and (2) there is merit in at least allowing for theoretical diversity.

First, Logan (1988) pointed out that behavior could be mediated by the similarity-based automatic retrieval of episodic representations from memory. Episodic memory traces differ from S-R associations as they are typically conceived of as being non-abstractive: whereas different experiences all contribute to the strength of a single association, episodic memory models assume that each individual experience is stored as a separate memory trace (e.g., Medin and Schaffer, 1978). Moreover, whereas simple associations do not specify the way in which events are related (e.g., whether A causes, predicts, or merely co-occurs with B; see Lagnado et al., 2007), episodic memory traces encode the way in which an event is constructed by an individual, including assumptions that are made about the relation between events. According to episodic models, stimuli in the current environment automatically activate episodic memory traces that contain information about similar stimuli. If those activated memory traces also contain information about a particular response, then this can lead to the automatic execution of that response. The likelihood that responses are automatically activated depends on the number of episodes that encompass both the stimulus and the response, as well as time that has elapsed since the episode was constructed. Hence, episodic models differ in important ways from S-R association formation models as they are typically conceived of (i.e., concrete vs. abstract; relational vs. associative; similarity-based retrieval vs. spreading of activation). Nevertheless, according to episodic models, a stimulus in the current environment can result in behavior that (1) has frequently been emitted in the context of that stimulus, (2) has features of automaticity, and (3) is merely stimulus-driven^5^. As such, episodic memory models such as those proposed by Logan (1988) provide an interesting alternative for S-R association formation models of habitual behavior.

Considering also episodic models of habitual behavior will increase theoretical diversity within the literature on habits, which is bound to enrich theoretical discussions and empirical research. For instance, unlike typical S-R association formation models, episodic memory models assign an important role to the recency of events and can thus inspire research that examines the relative contribution of frequency and recency in habitual behavior (see Schmidt et al., 2019, submitted, for an example). Moreover, because episodes can encode also instructions, episodic models might provide a new perspective on the finding that automatic behavior can result from simple instructions and implementation intentions (e.g., Martiny-Huenger et al., 2017; Meiran et al., 2017). Finally, because episodic models assign an important role to factors at retrieval, they can also inspire research on the context dependency of habitual behavior. With regard to the latter point, it remains to be seen whether the emphasis on retrieval factors in episodic models fits with what is known about the functioning of habit memory.

Second, habit researchers have recently benefited from another alternative for the traditional S-R association formation model, namely predictive coding models. These models have been highly influential in neurocognitive research on a variety of topics such as perception, memory, and attention (e.g., Friston, 2010, 2018; Clark, 2013). The core assumption is that organisms constantly build a mental model of the world which allows them to behave in ways that minimize energy expenditure. Both model construction and behavior selection are assumed to be based on inferential processes that can operate under conditions of automaticity. As such, predictive coding models provide a natural account of automatic behavior (Van Dessel et al., 2019). Stimulus-driven behavior, on the other hand, could be conceptualized as behavior that is guided by simple (i.e., hierarchically shallow) models that do not include information about higher order goals of the organism (see FitzGerald et al., 2014; Friston et al., 2016, for more details). Although predictive coding theories are not incompatible with the idea of gradually acquired S-R associations, they do provide a new perspective on how those S-R associations are formed and influence behavior. Moreover, considering predictive coding models could offer highly formalized theories of habits that allow for new predictions, for instance, with regard to what happens when habitual behavior does not lead to predicted outcomes (FitzGerald et al., 2014).

Note that considering alternative theories about habitual behavior does not change the fact that it is advisable to ban assumptions of specific mental representations and processes from the definition of habits. Regardless of the nature of the mental representations or processes that are assumed to be crucial for habits (S-R associations, episodes, predictive coding), it will always be difficult to verify empirically the involvement of a specific type of mental representation of process. Acknowledging multiple mental process explanations of habitual (in the sense of stimulus-driven) behavior also does not solve the problem that it is difficult to demonstrate with certainty that behavior is not directed at (hidden) goals. However, considering multiple theories about the mental representations and processes that mediate habitual behavior does enrich the theoretical debate and can thus lead to new discoveries.

## Final Thoughts on Overcoming the Challenges of Habit Research

Habit research is faced with many challenges (see de Wit et al., 2018; Watson and de Wit, 2018, for a discussion). The central message of this paper is that many of these challenges result from the inclusion of causal elements within the definition of habits. This not only makes it difficult to establish and thus study habits and habitual behavior (see section “The Empirical Level: Establishing the Presence of Habitual Behavior”) but can also constrain thinking about the mechanisms mediating habitual behavior (see section “The Theoretical Level: Explaining Habitual Behavior”). Hence, from this perspective, a possible solution for the challenges of habit research is to reduce the number of causal elements from the definition of habits and habitual behavior.

Based on the arguments presented above, it can be strongly recommended to remove any assumptions about S-R associations from the definition of habits and habitual behavior. Such assumptions aggravate the problems of empirically verifying the presence of habitual behavior and entail the risk of impoverishing theoretical debate by (implicitly or explicitly) committing researchers to *a priori* assumptions about the nature of the representations and processes that mediate stimulus-driven behavior. Theories about S-R associations can still be an important part of habit research but rather than being a part of the explanandum (i.e., that which needs to be explained) the role of these theories would be firmly restricted to that of one possible explanans (i.e., that which explains; Hempel, 1970).

What about the widespread idea that habits are stimulus-driven? As noted above, this idea introduces a number of causal elements within the definition of habits, not only about what is a cause of behavior (i.e., the stimulus) but also about what is not a cause of behavior (i.e., goals). Especially the latter element hampers the capacity to determine whether a behavior qualifies as habitual. However, as Gardner (2015) correctly pointed out, scientists are engaged with the causes of behavior rather than with simply describing behavior. As far as psychological explanations go, the distinction between explanations that do and do not involve goals is a fundamental one, not least because it has important implications for how to influence behavior (i.e., manipulating goals will only affect behavior that is mediated by those goals; De Houwer, 2019). Hence, it is understandable that cognitive scientists are interested in studying stimulus-driven behavior, that is, behavior that is not mediated by goals.

Nevertheless, researchers who wish to study habits in the sense of stimulus-driven behavior are well advised to proceed cautiously. The stimulus-driven nature of behavior cannot be observed directly, nor are there perfect proxies for establishing stimulus-driven behavior. As noted about, devaluation and contingency degradation test are strong indicators of the involvement of goals but not of the non-involvement of goals. Establishing the automaticity of behavior is not only difficult but also does not guarantee that the behavior is stimulus-driven. Although these difficulties should not stop researchers from examining stimulus-driven behavior, they need to be aware of these problems and take them into account when drawing conclusions.

Another option is to ban any reference to the stimulus-driven nature of habits from the definition of habits, which would leave only the notion that habits result from the frequent performance of a behavior in a certain context, as well as the notion that habits are automatic (Gardner, 2015). Choosing this option would imply that behavior is regarded as habitual if it can be established that (1) its presence is due to its past frequency and/or (2) it has features of automaticity. As noted above, both criteria are also not without problems. Establishing the role of frequency requires well-controlled experimental studies. Establishing the automaticity of behavior can entail many different, non-overlapping, and context-dependent automaticity features, some of which are difficult to verify because they refer to mental processes (Moors, 2016). Moreover, focusing exclusively on frequency would eliminate recency as a possible cause of habitual behavior. Hence, researchers who wish to define habit research as the study of frequent or automatic behavior should also be aware of the challenges entailed by this view on habits.

A possible way to reduce these challenges is to focus on features of automatic behavior that are relatively easy to verify. For instance, habit researchers could study behavior that is instigated quickly in certain contexts or that people subjectively experience as having little conscious control over. These criteria can be verified using experimental tasks or questionnaires. Once consensus over these criteria has been reached, researchers could document the moderators of those behaviors (i.e., the conditions under which behaviors with those automaticity features occur), which constrains theories about the mental mechanisms that produce those behavior. Such an approach would imply a clear separation between the explanandum of habit research (i.e., specific instances of automatic behavior) and the explanans of habit research (i.e., assumptions about the causal mechanisms that produce automatic behavior). It would also bring academic habit research closer to the notion that lay people have about habits.

Different researchers will probably choose different paths to overcome the challenges of habit research. Those whose primary interest lies in studying whether and when behavior is stimulus-driven (i.e., not mediated by goal representations) will probably continue to define habitual behavior as stimulus-driven behavior but, hopefully, ban any reference to specific S-R theories from their definitions, as well as use proxies of stimulus-driven behavior in a cautious manner. Those who wish to understand why behavior can be initiated quickly and why people sometimes report to have little control over their behavior will be probably be happy with defining habits as automatic behavior. The aim of this paper is not to convince researchers to ban all causal elements from the definition of habits (or other concepts in psychology), nor to promote a particular definition of habits. Instead, the main aim is to highlight that choosing a definition of habits has important implications for both empirical research (i.e., how to establish whether behavior is habitual) as well as theory development (i.e., proposals about the mechanisms that underlie habitual behavior). Hence, it is important to make explicit the causal assumptions that researchers make when using a particular definition of habits, as well as to acknowledge the challenges that these assumptions imply.

## Author Contributions

The author confirms being the sole contributor of this work and has approved it for publication.

### Conflict of Interest

The author declares that the research was conducted in the absence of any commercial or financial relationships that could be construed as a potential conflict of interest.

## Footnotes

^1^Note that the concept of stimulus-driven behavior does not overlap with the concept of respondent behavior that is often used by functional researchers (see Skinner, 1953). Like stimulus-driven behavior, respondent behavior is under the control of stimuli in the environment. However, unlike stimulus-driven behavior, behavior can be called respondent only if it was never before under the control of its consequences. This is an important distinction because it is typically assumed that many stimulus-driven behaviors are originally goal-directed but become stimulus-driven only as the result of the frequent execution of the behavior. Hence, most stimulus-driven behaviors do not qualify as respondent behavior. The distinction between respondent and stimulus-driven behavior is related to the fact that functional psychology focuses on functional causation (A is a function of B) whereas cognitive psychology focuses on mechanistic causation (A triggers B; see Chiesa, 1992, for an excellent discussion). Functional causation does not require contiguous causes (i.e., events in the here and now that put behavior in motion, much like one cogwheel can put another cogwheel in motion) but allows for causes that are present in the past. Hence, if the presence of a behavior in the past has been a function of its consequences (i.e., it was an operant behavior) and if its current presence is a function of its presence in the past (i.e., it is more likely now because it was repeatedly emitted in the past), then the current presence of the behavior is a function of the consequences of the behavior in the past, which is why also the current behavior would qualify as an operant behavior. The concept of stimulus-driven behavior, on the other hand, only takes into account contiguous causes and thus only entities that are present immediately before the behavior is initiated. For cognitive psychologists, these contiguous causes can be events in the current physical environment but also representations at the mental level. A behavior qualifies as stimulus-driven if the only contiguous cause of the behavior is (the representation of) a stimulus in the environment without the involvement of representations of goals. In sum, whereas the concept of respondent behavior is inherently functional in nature, the concept of stimulus-driven behavior is inherently mental in that it refers to the (absence of a) mechanistic causal impact of goal representations (see De Houwer, 2011; Hughes et al., 2016, for a discussion of the relation between functional and cognitive psychology). Within functional psychology, one could in principle study how the frequency of reinforcement in the past changes the moderators of behavior in the present (e.g., Barnes-Holmes et al., 2017).

^2^If one interprets “stimulus-driven” in a strict manner as indicating that the behavior is a function solely of the stimulus, then demonstrating the stimulus-driven nature of behavior would also require evidence that the behavior does not depend on any enabling conditions, such as the availability of sufficient attentional resources (Bargh, 1989). In this paper, however, I focus only on the assumption that goals are not causally involved in stimulus-driven behavior.

^3^Note that this problem in part arises because in this and many other studies with humans, researchers did not have full experimental control over the outcomes that at which behavior is directed. Instead, researchers often look at behaviors that were acquired before participants took part in the study (e.g., popcorn eating in cinema visitors). In most animal studies, on the other hand, the potentially habitual behavior has been established experimentally by linking it with a particular outcome (e.g., food). In these cases, there is more certainty about the outcome that is actually controlling the behavior during its initial stages. Hence, it is unlikely that the behavior is controlled by a different outcome when devaluation or contingency degradation tests suggest that it is no longer controlled by the original outcome of the behavior. Nevertheless, even in fully experimental research, one should take care that statistical power is sufficient to establish the absence of an effect (Vadillo et al., 2019) and that the devaluation and contingency degradation tests are sensitive enough.

^4^One could argue that the time needed to initiate or complete a behavior is related to whether the behavior can be considered a skill rather than to whether a behavior is considered to be a habit. Nevertheless, speed of performance has explicitly been put forward by some as a characteristic of habitual behavior, next to other automaticity features (e.g., Wood and Rünger, 2016, p. 292). Because of the difficulty in distinguishing conceptually and empirically between skills and habits, I will sidestep this issue in the current paper.

^5^Note, however, that episodes can also contain information about the goals that someone has when performing an action. Hence, automatic retrieval of episodes (and thus automatically activated behavior) could also depend on goals at the time of retrieval (Logan, 1988). In those cases, the behavior at the time of retrieval would not qualify as stimulus-driven. It would be interesting to run simulations to see whether there are circumstances in which goals at retrieval do not influence the automatic retrieval of episodes (e.g., when stimulus–response relations remain constant while goals vary). Such simulations could provide new insights into whether and when behavior is stimulus-driven.
